# Mating-induced reduction in accessory reproductive organ size in the stalk-eyed fly *Cyrtodiopsis dalmanni*

**DOI:** 10.1186/1471-2148-5-37

**Published:** 2005-06-09

**Authors:** David W Rogers, Tracey Chapman, Kevin Fowler, Andrew Pomiankowski

**Affiliations:** 1 The Galton Laboratory, Department of Biology, University College London, 4 Stephenson Way, London NW1 2HE, UK; 2 Collegium Budapest, Szentháromság utca 2, H-1014 Budapest, Hungary

## Abstract

**Background:**

Internal reproductive organ size is an important determinant of male reproductive success. While the response of testis length to variation in the intensity of sperm competition is well documented across many taxa, few studies address the importance of testis size in determining other components of male reproductive success (such as mating frequency) or the significance of size variation in accessory reproductive organs. Accessory gland length, but not testis length, is both phenotypically and genetically correlated with male mating frequency in the stalk-eyed fly *Cyrtodiopsis dalmanni*. Here we directly manipulate male mating status to investigate the effect of copulation on the size of both the testes and the accessory glands of *C. dalmanni*.

**Results:**

Accessory gland length was positively correlated with male mating frequency. Copulation induced a significant decrease in accessory gland size. The size of the accessory glands then recovered slowly over the next 8–48 hours. Neither testis length nor testis area was altered by copulation.

**Conclusion:**

These results reveal that the time course of accessory gland recovery corresponds to field observations of mating behaviour and suggest that accessory gland size may limit male mating frequency in *C. dalmanni*.

## Background

There is a considerable body of evidence that reproductive organ size contributes to male reproductive success. This mainly derives from interspecific comparisons that have found positive relationships between testis size and the risk of sperm competition [1-5]. In addition, the direct manipulation of sperm competition intensity under experimental evolution has been shown to cause correlated changes in testes size in two species of Diptera [6,7]. However, few studies have addressed the importance of internal reproductive organ size to other components of male reproductive success, or the significance of size variation in accessory reproductive organs which are often vital for sperm transfer, fertility, and essential for success in sperm competition [8,9].

In this paper, we investigate how reproductive organ size may limit male mating frequency under conditions where males encounter high numbers of mating opportunities and are thus potentially at risk of sperm or seminal fluid depletion [10-12]. Previous data support the hypothesis that male mating frequency can be limited by reproductive organ size in insects. For example, in dung flies, the length of the proximal section of the testis decreases with the number of copulations achieved in *Scathophaga stercoraria *[13] and increasing copula duration in *Sepsis cynipsea *[14]. Testis mass is also lower in mated than in unmated Dawson's burrowing bees *Amegilla dawsoni *[15]. In contrast, accessory gland size, but not testis size, is phenotypically correlated with male mating frequency in *Drosophila melanogaster *[16] and accessory glands become completely depleted and reduced in volume after 4–5 matings, leading to decreased fertility even though motile sperm remain in the seminal vesicles [17,18]. The ability to replenish reserves of sperm and seminal fluid likely further constrains male mating frequency (reviewed in [10]). Mating stimulates the replenishment of accessory gland products in *D. melanogaster *[19]. This resynthesis reaches a maximum after 2–4 hours and decreases to basal levels after 48 hours in *Drosophila funebris *[20].

In this study, we used the stalk-eyed fly *Cyrtodiopsis dalmanni *to test whether testis and accessory gland size are affected by mating. This is an ideal species, as males and females regularly mate at extremely high frequency [21-23]. Over 90% of matings occur in nocturnal aggregations which usually consist of a single male and a harem of several females [24] (up to 24 in the closely related species *Cyrtodiopsis whitei *[25]). Females join aggregations each evening and mate in the period immediately following dawn before dispersing [25,26]. During copulation, males transfer a single small spermatophore composed of sperm from the testes enveloped in accessory gland secretions [27]. Previous work has shown that accessory gland length, but not testis length, is phenotypically correlated with male mating frequency [22]. Additionally, bidirectional artificial selection on male mating frequency resulted in a correlated response in accessory gland length but not in testis length [23]. While correlative evidence, whether phenotypic or genetic, indicates an association between accessory gland size and male mating frequency, it does not establish a direct physiological relationship between these two variables. In the current study, we provide direct evidence that mating induces a decrease in accessory gland, but not testis, size. Furthermore, we demonstrate that the timecourse of post-copulatory recovery of accessory gland size closely mirrors field observations of mating patterns in *C. dalmanni*.

## Results

**Figure 1:**
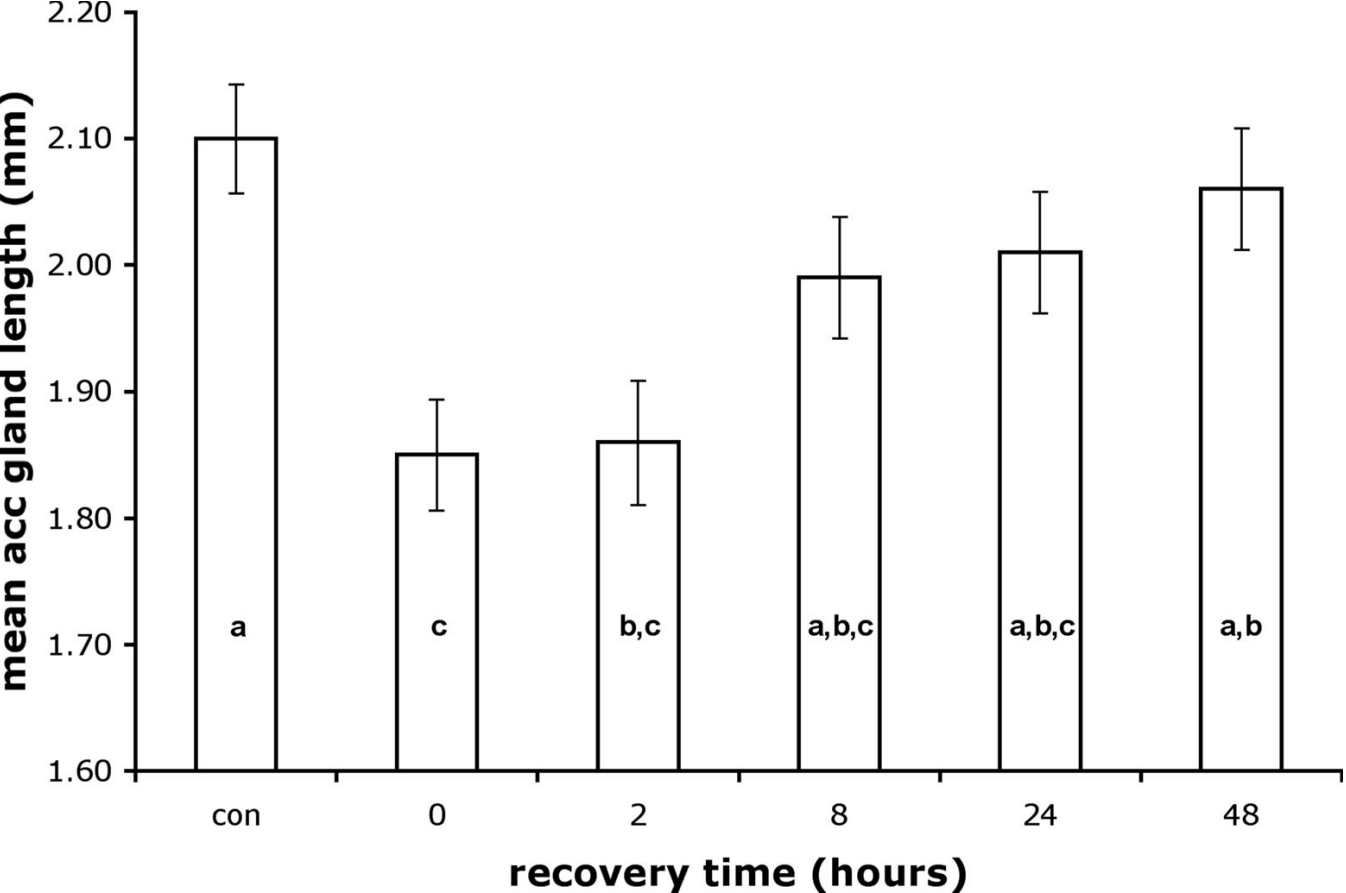
**Reduction and subsequent recovery of accessory gland length following mating. **Mean accessory gland length decreased from 2.10 mm to 1.85 mm following mating and was restored to the original size within 8–48 hours. Controls (con) were unmated (virgin) males. Columns not marked with the same letter are significantly different (Tukey HSD). Values shown are least squares means ± s.e. at average values of male eyespan.

We manipulated male mating status by providing males with the opportunity to mate with 6 virgin females for 60 minutes immediately following artificial dawn. Mated males were dissected at fixed times following this mating period (0 hours, 2 hours, 8 hours, 24 hours and 48 hours) and the sizes of their testes and accessory glands were compared to unmated control males. Mating resulted in a significant decrease in accessory gland length, but glands returned to their original size over the course of the next 8 to 48 hours. At average levels of male eyespan, included to as a measure of body size to control for allometric variation (*F*_1,185 _= 5.25, *p *= 0.0231), mating status affected accessory gland length (*F*_5,185 _= 4.72, *p *= 0.0004). Post-hoc Tukey HSD tests revealed that males dissected immediately after mating or 2 hours after mating exhibited significantly smaller accessory glands than unmated controls. Gland length began to recover after 8 hours and by 48 hours after mating the accessory glands were significantly longer than immediately following mating (Fig. 1). Removing unmated control males from the analysis revealed a positive effect of mating frequency on accessory gland length (*b *± s.e. = 0.0228 ± 0.0086, *t*_149 _= 2.67, *p *= 0.0085) after controlling for the significant effect of recovery time (*F*_4,149 _= 3.38, *p *= 0.0111). Males mated a mean ± s.e. of 3.79 ± 0.20 (range: 1–12) times during the course of the 60 minute observation period, and mating frequency did not vary between groups dissected at different times *F*_4,150 _= 1.08, *p *= 0.3667). Identical results were obtained when accessory gland length was replaced with area, but are not included as accessory gland length and the square root of area were highly positively correlated (r_90 _= 0.926, *p <*0.0001).

**Figure 2:**
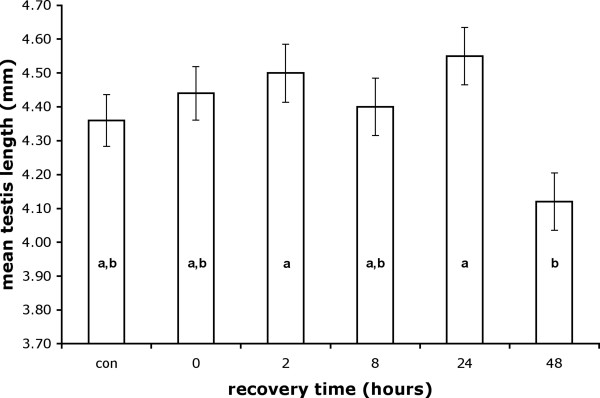
**Response of testis length to mating. **Males dissected 48 hours after mating exhibited smaller testes than males dissected at 2 hours and 24 hours post-mating. Controls (con) were unmated (virgin) males. Columns not marked with the same letter are significantly different (Tukey HSD). Values shown are least squares means ± s.e. at average values of male eyespan.

Mating did not result in a decrease in testis length compared to unmated controls (Tukey HSD, Fig. 2). However, significant differences in testis length were detected between males measured at different recovery times (*F*_5,185 _= 3.10, *p *= 0.0102). Post-hoc Tukey HSD tests revealed that males allowed to recover for 48 hours exhibited shorter testes than males allowed to recover for 2 or 24 hours. Testis length scaled with male eyespan (*F*_1,185 _= 1.71, *p *= 0.0054). Removing unmated males from the analysis failed to reveal any association between testis length and mating frequency (*F*_1,147 _= 0.68, *p *= 0.4100) after controlling for recovery time *F*_4,147 _= 3.66, *p *= 0.0071) and eyespan (*F*_1,147 _= 4.33, *p *= 0.0392). Testis length and the square root of area were positively correlated (r_61 _= 0.691, *p *< 0.0001). As testis length explained less than half of the variance in testis area (r^2 ^= 0.477), we also directly compared testis area in males immediately after mating to that in unmated controls and detected no difference (mean ± s.e.: mated = 0.801 ± 0.021, unmated = 0.792 ± 0.020, *t*_65 _= 0.276, *p *= 0.7834).

## Discussion

Male accessory gland size in *C. dalmanni *decreased dramatically following copulation and slowly recovered over the next 8–48 hours. After removing the effect of recovery time, accessory gland length was positively correlated with male mating frequency. Neither testis length nor testis area appeared to be altered by copulation; no significant difference in testis length was observed between mated and unmated males in the 48 hours following copulation.

Both male and female stalk-eyed flies mate frequently. In the current study, each male mated an average of 3.79 times (up to a maximum of 12) during the 60-minute observation period. Only 23.9% (37 out of 155) of males mated at least 6 times and therefore 76.1% (118 out of 155) of males failed to mate with all 6 virgin females provided. As females housed with three males will mate an average of 5.51 times during the 60 minutes following artificial dawn [28], it is clear that male mating frequency was limited by physiological ability rather than the availability of willing females.

In the field, copulations occur primarily at dawn [24,25]. Our observations of the recovery of the accessory glands match this behavioural pattern, as 24 hours after copulation (i.e. the subsequent dawn period), the accessory glands had recovered their original pre-mating size. We found that the accessory glands had partially recovered after 8 hours which is consistent with the lower frequency of mating observed at dusk [24,25], whereas little recovery was observed in the hours immediately following copulation when flies leave mating aggregations to forage.

The two most plausible physiological constraints on male mating frequency in *C. dalmanni *are the availability of accessory gland products and the availability of sperm, both of which are required to produce spermatophores [27]. Several lines of evidence indicate that accessory gland size is more likely to limit mating frequency than testis length. First, we have demonstrated a decrease in accessory gland size following copulation and the subsequent recovery closely mirrors mating behaviour in the field. No significant reduction in testis size was observed in mated males compared to unmated controls. Second, our study confirms the results of a previous experiment showing that accessory gland length, but not testis length, is phenotypically correlated with male mating frequency [22]. Third, bidirectional artificial selection on male mating frequency produced a correlated response in accessory gland length but not testis length [23]. However, we cannot exclude the possibility that some other currently unknown factor is the primary constraint on male mating frequency in *C. dalmanni*.

The full importance of the accessory glands in stalk-eyed fly reproduction is poorly understood. Accessory gland products form the casing of the spermatophore and consequently are necessary for sperm transfer [27]. Furthermore, accessory gland products appear to be important in sperm competition as seminal fluid can decrease the viability of sperm from particular rival males in the female spermathecae [29]. However, in contrast to *D. melanogaster *[30], accessory gland products do not appear to play a role in sperm displacement [29], the inhibition of female remating [31] or the manipulation of female fecundity [28]. Consequently, the advantage of large accessory glands is likely gained through both increased mating frequency (by allowing males to produce more spermatophores over a given time period) and, potentially, greater success under sperm competition.

## Conclusion

When receptive females are not limiting, male mating frequency in *C. dalmanni *is likely constrained by accessory gland size. Copulation causes a significant reduction in accessory gland size and replenishment of the depleted accessory glands follows a time course that is consistent with the observed daily peak in male mating frequency at dawn. There was no reduction in testis size following mating and therefore testis size appears to be of less importance in determining male mating frequency in this species.

## Methods

### General methods

The base stock was an outbred laboratory population of the stalk-eyed fly, *C. dalmanni*, collected from Gombak, Malaysia in 1993. The stock was maintained in large cages at high population size (typically more than 200 individuals per cage) and with a 1:1 sex ratio. Flies were fed ground corn medium and kept at 25°C on a 12 h/12 h light/dark regime. The regime included a 15-min "dawn" period in which the culture room was illuminated by a single 60-W bulb. All observations of behaviour commenced at the start of this dawn period.

### Manipulation of male mating status

Experimental flies were raised from eggs collected in groups of 13 from the population cages and allowed to hatch on moist cotton pads in Petri dishes containing at least 2 g of ground corn (maize). Upon eclosion, flies were segregated according to sex and raised to sexual maturity in groups of 10 housed in 1.5 L plastic pots on an *ad libitum *diet of ground corn. Mating observations were conducted using virgin males aged 6 weeks post-eclosion and virgin females aged 6–8 weeks post-eclosion. Males were randomly assigned to 5 mating status groups: unmated controls (n = 36), 0 hours recovery (n = 38), 2 hours recovery (n = 29), 8 hours recovery (n = 30), 24 hours recovery (n = 30), and 48 hours recovery (n = 30). At artificial dawn, individual males were added to 1.5 L plastic pots containing 6 females, except for control males which were placed in empty 1.5 L pots. The number of copulations over 40 seconds in duration occurring during the subsequent 60-minute period was recorded. Males that failed to mate during this observation period were discarded. Unmated control males and 0 hour recovery males were immediately placed on ice and dissected. Males assigned to other recovery periods were moved individually to 500 ml plastic pots lined with a moist cotton pad and provided with ground corn until the appropriate time of dissection.

### Morphological measurements

Males were dissected in a small amount of phosphate buffered saline on a microscope slide. Images of the accessory glands and uncoiled testes were captured using a monocular microscope connected via a video camera to a Macintosh computer with NIH Image (version 1.55). Length was measured by tracing a midline that longitudinally bisected each organ and the mean length of the two accessory glands or testes was used in analyses. Area was measured by tracing the outline of each organ and calculating the longitudinal surface area. Areas of both accessory glands were calculated and the mean used in analyses, but a single randomly chosen testis was measured per individual. Eyespan, was defined as the distance between the outer tips of the eyes.

### Statistical analyses

Unless otherwise indicated, general linear models were used to analyse the determinants of reproductive organ size. Initial models included an intercept, male eyespan, recovery time and the eyespan × recovery time interaction. Recovery time was coded into models as an ordinal categorical variable. Stepwise elimination was used to remove terms that failed to significantly improve the fit of the model. Secondary analyses extended the models to include the number of copulations observed which required the exclusion of control males that did not copulate. Data sets did not deviate significantly from the assumptions of general linear modelling.

## Authors' contributions

DWR conceived of the study, contributed to the design, carried out the experimental work and statistical analysis, and drafted the manuscript. TC, KF, and AP participated in the design and coordination of the study and helped to draft the manuscript. All authors read and approved the final manuscript.

## References

[B1] Harcourt AH, Harvey PH, Larson SG, Short RV (1981). Testis weight, body weight and breeding system in primates. Nature.

[B2] Møller AP (1991). Sperm competition, sperm depletion, paternal care, and relative testis size in birds. Am Nat.

[B3] Gage MJG (1994). Association between body size, mating pattern, testis size and sperm lengths across butterflies. Proc R Soc Lond B.

[B4] Hosken DJ (1997). Sperm competition in bats. Proc R Soc Lond B.

[B5] Stockley P, Gage MJG, Parker GA, Møller AP (1997). Sperm Competition in fishes: the evolution of testis size and ejaculate characteristics. Am Nat.

[B6] Pitnick S, Miller GT, Regan J, Holland B (2001). Males' evolutionary responses to experimental removal of sexual selection. Proc R Soc Lond B.

[B7] Hosken DJ, Ward PI (2001). Experimental evidence for testis size evolution via sperm competition. Ecol Lett.

[B8] Leopold RA (1976). The role of male accessory glands in insect reproduction. Annu Rev Entomol.

[B9] Gillot C (2003). Male accessory gland secretions: modulators of female reproductive physiology and behavior. Annu Rev Entomol.

[B10] Dewsbury DA (1982). Ejaculate cost and male choice. Am Nat.

[B11] Cartar RV (1985). Testis size in sandpipers. Naturwissenschaften.

[B12] Preston BT, Stevenson IR, Pemberton JM, Wilson K (2001). Dominant rams lose out by sperm depletion. Nature.

[B13] Ward PI, Simmons LW (1991). Copula duration and testes size in the yellow dung fly, *Scathophaga stercoraria *(L.): the effects of diet, body size, and mating history. Behav Ecol Sociobiol.

[B14] Martin OY, Hosken DJ (2002). Strategic ejaculation in the common dung fly *Sepsis cynipsea*. Anim Behav.

[B15] Simmons LW, Tomkins JL, Alcock J (2000). Can minor males of Dawson's burrowing bee, *Amegilla dawsoni *(Hymenoptera: Anthophorini) compensate for reduced access to virgin females through sperm competition?. Behav Ecol.

[B16] Bangham J, Chapman T, Partridge L (2002). Effects of body size, accessory gland and testis size on pre- and postcopulatory success in *Drosophila melanogaster*. Anim Behav.

[B17] Lefevre G, Jonsson UB (1963). Sperm transfer, storage, displacement, and utilization in *Drosophila melanogaster*. Genetics.

[B18] Hihara F (1981). Effects of the male accessory gland secretion on oviposition and remating in females of *Drosophila melanogaster*. Zool Mag.

[B19] Herndon LA, Chapman T, Kalb JM, Lewin S, Partridge L, Wolfner MF (1997). Mating and hormonal triggers regulate accessory gland gene expression in male *Drosophila*. J Insect Physiol.

[B20] Baumann H (1974). The isolation, partial characterization, and biosynthesis of the paragonial substances, PS-1 and PS-2, of *Drosophila funebris*. J Insect Physiol.

[B21] Baker RH, Ashwell RIS, Richards TA, Fowler K, Chapman T, Pomiankowski A (2001). Effects of multiple mating and male eye span on female reproductive output in the stalk-eyed fly *Cyrtodiopsis dalmanni*. Behav Ecol.

[B22] Baker RH, Denniff M, Futerman P, Fowler K, Pomiankowski A, Chapman T (2003). Accessory gland size influences time to sexual maturity and mating frequency in the stalk-eyed fly, *Cyrtodiopsis dalmanni*. Behav Ecol.

[B23] Rogers DW, Baker RH, Chapman T, Denniff M, Pomiankowski A, Fowler K (2005). Direct and correlated responses to artificial selection on male mating frequency in the stalk-eyed fly *Cyrtodiopsis dalmanni*. J Evol Biol.

[B24] Wilkinson GS, Reillo PR (1994). Female choice response to artificial selection on an exaggerated male trait in a stalk-eyed fly. Proc R Soc Lond B.

[B25] Lorch PD, Wilkinson GS, Reillo PR (1993). Copulation duration and sperm precendence in the stalk-eyed fly *Cyrtodiopsis whitei *(Diptera: Diopsidae). Behav Ecol Sociobiol.

[B26] Burkhardt D, de la Motte I (1987). Physiological, behavioural, and morphometric data elucidate the evolutive significance of stalked eyes in Diopsidae (Diptera). Entomol Gen.

[B27] Kotrba M (1996). Sperm transfer by spermatophore in Diptera: new results from Diopsidae. Zool J Linn Soc.

[B28] Reguera P, Pomiankowski A, Fowler K, Chapman T (2004). Low cost of reproduction in female stalk-eyed flies, *Cyrtodiopsis dalmanni*. J Insect Physiol.

[B29] Fry CL, Wilkinson GS (2004). Sperm survival in female stalk-eyed flies depends on seminal fluid and meiotic drive. Evolution.

[B30] Wolfner MF (2002). The gifts that keep on giving: physiological functions and evolutionary dynamics of male seminal proteins in *Drosophila*. Heredity.

[B31] Grant CA, Fowler K, Chapman T (2002). No reduction of female sexual receptivity following mating in a stalk-eyed fly, *Cyrtodiopsis dalmanni *(Diptera: Diopsidae). J Evol Biol.

